# Personalized medicine for schizophrenia

**DOI:** 10.1038/s41537-016-0001-5

**Published:** 2017-01-05

**Authors:** Peter F. Buckley, Brian J. Miller

**Affiliations:** Medical College of Georgia, Augusta University, 1120 15th Street, AA-1002, Augusta, GA 30912 USA

A provocative psychopharmacology paper from an earlier era,^1^ purporting to show bias in clinical trials for schizophrenia, actually elicits a more sobering interpretation—namely, that the treatment of patients with schizophrenia is no better than a ‘trial-and-error’ approach for each patient with each drug. For sure, it’s “individualized” or “personalized” treatment—to the extent that treatment (and lesser so adverse effects) is highly variable across each patient - though this is certainly a long way off from the genetically-guided immunotherapy and individually tailored cancer therapy that characterizes personalized medicine (PM) as having come of age in healthcare and science.^2–5^ Moreover, biomarker tests are emerging across several areas of medicine that inform and guide molecular targeted therapies that have transformative potential for how we deliver care.

In a thoughtful appraisal of PM, Jameson and Longo^6^ define PM as “treatments targeted to the needs of individual patients on the basis of genetic, biomarker, phenotypic, or psychosocial characteristics that distinguish a given patient from other patients with similar clinical presentations.” The extent to which this promise will arrive at the doorsteps of psychiatry and redefine our treatment for schizophrenia is presently uncertain;^7–10^ we remain challenged by fundamental nosological issues,^11^ absence of a clear and underlining neurobiology for schizophrenia. Some 40 years after the notorious “pink spot” (an earlier fanciful urine test that was considered pathognomic for schizophrenia!), there are a plethora of neurobiological measures from genetics, “OMICS” (proteomics, lipidomics, and metabolomics), electrophysiology, and brain imaging (including multiple structural and functional modalities) that have yielded discriminatory findings between patients with schizophrenia and control subjects and often—but by no means invariably—between patients with schizophrenia and mood disorders.^7,10^ All that said, the ability is not yet there for a single measure—or even a collective complement of measures—to reliably discriminate as a biomarker for diagnosis and/or treatment in schizophrenia. Biomarkers, despite encouraging findings across disparate measures and study populations, are not quite “ready for primetime”. As an exemplar of progress made to date, Clark *et al*.^12^ report an accuracy (with 72.7% sensitivity, 96.4% specificity) of a battery of tests predicting a transition to psychosis among youth at high risk for psychosis. Others report similar findings,^13^ and clearly if we could “diagnose” schizophrenia in pre-symptomatic people—and intervene accordingly—that would be a game changer.

Another early, yet nevertheless encouraging finding is of the identification of some 108 at risk genes associated with schizophrenia, with apparent overlap in areas as related to calcium-channel regulation and immunological markers.^14^ This work points to a potentially fertile area of neuroimmunology of schizophrenia.

As stated earlier, despite many robust pharmacogentic studies, treatment selection remains a joint decision by patient and doctor, based more on intuition and experience than on any biological distinction.^9^ This is highly problematic. There is some (potential) light at the end of the tunnel by way of ever-increasing more diverse pharmacological design and receptor affinity of putative antipsychotic drugs. This offers the opportunity to at least determine mechanistically distinct groups of patients that might preferentially respond to one drug or another. As this work proceeds, our field is also hampered by the considerable challenge of selecting and including appropriate biomarkers in clinical trials of sufficient numbers of patients to detect biologically derived responses to treatment.

Across a broader scientific and political landscape, our field is converging on the strategic prioritization of the National Institute of Health and its subcomponent, the National Institute of Mental Health.^15^ Our field will also need to take stock of the directions of “convergence science”, population health and information technology analytics (so called “big data”), and of the moderating influences of social determinants of health and diversity among our patient populations.^16^

PM is already common parlance in other areas of medicine. For schizophrenia, the promise is still some way off. The need is great.
